# Cell therapy in pediatric blood diseases

**DOI:** 10.3389/fmed.2025.1591287

**Published:** 2025-06-25

**Authors:** Rubí Romo-Rodríguez, Rosana Pelayo, Dalia Ramírez-Ramírez

**Affiliations:** ^1^Laboratory of Oncoimmunology and Cytomics of Childhood Cancer, Centro de Investigación Biomédica de Oriente, Instituto Mexicano del Seguro Social, Puebla, Mexico; ^2^Secretaría de Ciencia, Humanidades, Tecnología e Innovación (Secihti), Mexico City, Mexico; ^3^Unidad de Educación e Investigación, Instituto Mexicano del Seguro Social, Mexico City, Mexico

**Keywords:** pediatric hematological diseases, HSC transplantation, MSC transplantation, CAR-T cells, CAR-NK cells

## Abstract

The fight against mortality in pediatric oncohematological diseases is a story of human rights, academia, innovation, and technological advancement—one in which women in science have played a pivotal role. With talent, perseverance, and sensitivity, they greatly contributed to research in new cellular therapies, expanding the frontiers of treatment and giving the most vulnerable patients the gift of more years of life. This review explores multipotent and unipotent cell therapies, focusing on hematopoietic stem cell transplantation (HSCT), mesenchymal stromal cell transplantation (MSCT), and chimeric antigen receptor (CAR)-T cell and natural killer (NK) cell therapy. HSCT remains the gold standard for high-risk and relapsed cases, with graft sources including bone marrow (BM), mobilized peripheral blood (MPB), and umbilical cord blood (UCB). Advances in MSCT highlight its role in hematopoiesis support and immunomodulation, reducing graft-versus-host disease (GVHD) risks. CAR-T cell therapy has revolutionized leukemia treatment, although challenges such as antigen escape, T-cell exhaustion, and treatment resistance persist. Emerging strategies, including CAR-NK cells, seek to enhance efficacy while minimizing toxicity. Despite these advancements, cell therapy remains complex and resource-intensive, necessitating further innovations for broader accessibility, particularly in developing regions.

## Introduction

In recent years, cell therapy procedures have steadily increased worldwide (1, 2). This treatment involves transferring viable cells into a patient as a treatment for various diseases (3). Here, we highlight the major contributions of women in science dedicated to pediatric cancer research.

Cell therapy is recommended for various pediatric hematologic conditions and utilizes patients-derived or donor cells to target malignant cells more effectively than chemotherapy and radiotherapy. It is classified as either autologous, using the patient’s own cells, or allogeneic, using human leukocyte antigen (HLA)-match or haploidentical donor cells, who can be related or unrelated to the patient (4). Another classification distinguishes multipotent stem cells, such as hematopoietic stem cells (HSCs) and mesenchymal stromal cells (MSCs), which are immature cells capable by long-term self-renewal, quiescence, and differentiation into multiple cell types (5), and unipotent cell therapies, which use mature, highly specialized cells that may undergo further modification before reintroduction into the patient, examples include CAR-T and CAR-NK cells (3).

## Multipotent stem cells therapy

### Hematopoietic stem cell transplantation (HSCT)

Hematopoiesis produces billions of blood cells daily, initiated by HSCs that differentiate into lymphoid and myeloid lineages. This process is tightly regulated by internal and external signals within BM niches (6). HSCT, the primary cellular therapy (7), replaces the recipient’s hematopoietic system (4) using HSCs from BM, MPB, or UCB (8).

In autologous HSCT, HSCs restore hematopoiesis after toxic therapies, while in allogeneic HSCT, the donor immune system significantly contributes to the elimination of malignant cells via the graft-versus-leukemia (GVL) effect (4, 9). Alloreactive donor T cells exert a powerful GVL effect, because they recognize host alloantigens on leukemic cells and kill them but also can cause GVHD, a severe complication affecting non-hematopoietic tissues (such as skin, gut, and liver). Matching HLA between recipient and donor is crucial to lower the risk of GVHD (10). Pediatric indications for allogeneic HSCT include acute lymphoblastic leukemia (ALL), acute myeloid leukemia (AML), and aplastic anemia (AA), while autologous HSCT is mainly used for neuroblastoma (a non-hematological malignancy) and Hodgkin lymphoma (HL) (1).

Bone marrow was the first HSCs source for transplants (11), but its invasiveness led to MPB emerging as the best alternative for donors. MPB involves mobilizing hematopoietic progenitor and stem cells (HPSCs) from BM to the peripheral blood, followed by leukapheresis (11, 12). UCB is valuable for patients without HLA-matched donors (13), offering greater HLA flexibility, with successful transplants occurring even with up to 3/6 mismatches (11). However, UCB transplants face challenges, including delayed engraftment, higher graft failure risk, and slower immune recovery due to the lower HPSCs doses (14). To compensate, double UCB transplants infuse two partially HLA-matched units (13), but typically, only one dominates and contributes to hematopoiesis (11). While double UCB transplants reduced relapse risk (RR 0.57, 95% CI 0.38–0.88), single UCB showed better overall survival (OS) (RR 1.25, 95% CI 1.06–1.46), with no major differences in chronic GVHD, non-relapse mortality, or leukemia-free survival (13). Over recent years, the use of UCB grafts has decreased (14), and BM remains the primary graft source for pediatric patients (1).

Advances in treatment strategies, including HSCT, have led to survival rates for childhood ALL and AML now reaching 90% and 70%, respectively – percentages reported in high-income countries, specifically the United States (15, 16). For high-risk or relapsed cases, allogeneic HSCT represents the best curative option (13, 15, 16). ALL outcomes remain poorer in adolescents and young adults (AYA). A study in Japan found that after HSCT, AYA patients had a lower 5 years OS rate and higher treatment-related mortality (TRM) than children, mainly due to infections. Relapse rates were similar across ages (22%–25%) (15). T-cell ALL (T-ALL), comprising 15% of pediatric ALL, is high risk, with a 33.82% relapse rate post-HSCT. Measurable residual disease (MRD) below 0.01%, adequate CD34+ cell infusion, chronic GVHD, or cytomegalovirus reactivation may provide protective effects against relapses in pediatric HSCT recipients with T-ALL (17). In pediatric AML, HSCT is recommended in first complete remission (CR) for high-risk cases. A study excluding acute promyelocytic leukemia found superior long-term survival in MRD-undetectable patients, regardless of whether HSCT was performed in CR1 or CR2, conditioning regimen, donor type, or genetic rearrangement. Additionally, HSCT in CR2 was independently associated with a worse outcome (16).

Relapse is the leading cause of death after 100 days post-HSCT (1). Timely immune reconstitution enhances the GVL effect (18). NK cells, the first donor-derived lymphocytes to recover, are promoted by the interleukin-15 (IL-15) rich cytokine milieu post-transplant, they help control residual malignant cells and viral infections. NK cell recovery occurs within 3–6 months (19). NK cell reconstitution is delayed in pediatric patients with acute or chronic GVHD, with acute GHVD patients showing lower levels of CD56^bright^ NK cells (20). In a pediatric cohort CD3+/CD4+/CD8+ lymphocyte counts at day 100 post-HSCT and acute GVHD had the greatest impact in preventing relapse. Immune reconstitution is influenced by recipient age, donor type (matched/mismatched), graft composition, conditioning regimen, acute GvHD, its treatment (e.g., steroid administration), prophylaxis, and infections (bacterial, viral, or fungal) (18).

Hematopoietic stem cell transplantation is a critical rescue therapy for high-risk or relapse cases; however, its implementation is complex, requiring highly trained personnel, advanced equipment, and adequate infrastructure. Consequently, access to these facilities remains limited in developing countries (21).

### Mesenchymal stromal cells transplantation (MSCT)

Mesenchymal stromal cells are multipotent cells capable of differentiating into adipocytes, osteocytes, chondrocytes, and other connective tissues cells (22). They regulate HSC proliferation and differentiation within the BM niche, supporting normal hematopoiesis (23), while also exhibiting immunomodulatory properties (24) and aiding tissue repair (25). MSCs can be isolated from BM, umbilical cord, adipose tissue, peripheral blood, liver and tooth root. They can be cultured *in vitro* and transplanted for therapeutic applications (22, 23). Though BM is the primary MSCs source, its collection is invasive. UCB and placenta have demonstrated comparable abilities to increase the proliferation and expansion of hematopoietic progenitor cells, with placental yielding higher MSCs quantities than UCB. Additionally, BM-derived MSCs decline in number, proliferation and differentiation capacities with age (26).

Mesenchymal stromal cells co-transplantation has shown promise in enhancing HSCT outcomes by stimulating hematopoiesis and regenerating stromal cells after chemotherapy. This approach accelerates T cell recovery in patients with low HSCs input undergoing autologous HSCT for malignant lymphomas (27). In allogeneic HSCT, the BM niche is critical for donor HSCs engraftment and reconstitution potential, long-term damage to the BM niche results in a reduced presence of proliferation-capable MSCs and poorer clinical outcomes (28).

Allogeneic MSCs are safe to infuse due to their intermediate HLA class I expression and lack of HLA class II molecules. MSCs evade alloreactive recognition, this advantage can be used allowing their storage for immediate use when needed, as they are not targeted for destruction by cytotoxic T lymphocytes (CTLs) or NK cells (29). The chemokine CXCL12, which plays a role in cell migration and homing, is upregulated in injured tissues, recruits MSCs via its receptor, CXC chemokine receptor 4 (CXCR4) (25). A promising strategy to enhance MSC homing to BM has been evaluated in preclinical studies. In a mouse model, infusion of MSCs overexpressing CXCR4 significantly increased leukocyte, erythrocyte, hemoglobin, and platelet counts. MSCs promote hematopoietic recovery and modulate the Treg/T17 balance in AA (25). A meta-analysis showed MSCs co-transplantation promotes neutrophil and platelet engraftment, reduces chronic GVHD, without increasing mortality or relapse rates, making MSCs an ideal treatment for pediatric HLA-mismatch HSCT (22). Importantly, MSCs inhibit T cells during the early activating phase of allograft reactions but not in the effector phase. They also secrete cytokines such as IL-11, IL-12, and IL-15, which may sustain a GVL effect without inducing GVHD (29).

## Unipotent cells therapy

### CAR-T cells

Chimeric antigen receptors (CARs) direct cells to a chosen antigen and reprogram the function, metabolism, and persistence of T cells independently of the major histocompatibility complex (MHC) (30). The first generation of CAR-T cells contain only the CD3-ζ intracellular domain, responsible for the initial activation of the T cell (31). A costimulatory domain, CD28 or 4-1BB, was added to CD3-ζ for the second generation to improved T cell proliferation after repeated exposure to antigen, cytokine secretion, and resistance to apoptosis, increasing their therapeutic efficacy (30, 31). In the third generation, combinations of multiple costimulatory molecules such as CD28-4-1BB or CD28-OX40 were added. Additionally, the complementary co-stimulation provided by the 4-1BB and CD28 endodomains from the CAR and CCR (chimeric costimulatory receptor) combination conferred increased cytokine secretion and expansion to improved persistence *in vivo* (32). Fourth-generation CAR-T cells, also known as TRUCKs (CAR-T cell redirected for universal cytokine killing), are engineered to express cytokines as IL-12 or IL-18 (33). This leads to improved cell function while also modulating the tumor microenvironment (TME) (34).

The introduction of CAR-T cells has transformed the treatment of cancer, significantly improving early response rates in pediatric ALL (35). Between 2016–2021, the 3 years survival rate for pediatric ALL patients in the United States after their first CAR-T cells treatment was 67.9% (1). Tisagenlecleucel was the first CAR-T cell therapy approved by the FDA in 2017 for the treatment of ALL in patients under 25 years of age (36). Also, the combination of CAR-T cells followed by alloHSCT on CAYAs can achieve durable disease control in many cases of relapsed or refractory (r/r) B-ALL (37).

Due to the cytokine release syndrome (CRS) and neurotoxicity associated with Tisagenlecleucel, new second generation CAR-T therapies are being developed. Brexucabtagene autoleucel (ZUMA-4 clinical trial, NCT02625480) (38, 39), Axicabtagene ciloleucel (NCT03642699) (38), and Lisocabtagene maraleucel (NCT03743246) are currently under evaluation in pediatric patients aged ≤ 25 years (40) with (r/r) B-ALL or r/r B-cell non-HL. Among them, positive outcomes have been reported in pediatric patients enrolled in the ZUMA-4 trial treated with Brexucabtagene autoleucel, with an overall response rate of 67%. Grade 3 CRS occurred in 33% of patients, and neurotoxicity was observed in 67%; however, all patients recovered (38).

Until the FDA approves these new therapies, Tisagenlecleucel has been shown to be a safe and effective option also in atypical pediatric populations with B-ALL, such as patients with central nervous system infiltration, early relapse, persistent MRD, high-risk genetic alterations, and Down syndrome (41, 42). Data supports its use beyond the initial indications (41), emphasizing the need to improve equitable access to patients who were not initially eligible and its long-term evaluation.

However, several mechanisms of resistance to CAR-T cell immunotherapy have been described, including direct CAR-T cell dysfunction, tumor-intrinsic mechanisms, and the immunosuppressive TME (43). Dysfunctional T cells can be exhausted (32, 44–46) or senescent (43, 47) revealing lower expansion capacity, reduced cytotoxic function and higher expression of inhibitory receptors. Various approaches have been developed to reduce exhaustion and senescence such blockade of PD1/PDL1 axis, PD1 and TIGIT, TGF-β, depletion or knockout of TOX/NR4A and the use of CAR-NK cells (43, 48). CD28-costimulated CARs expand quickly and secrete higher levels of inflammatory cytokines but have limited persistence due to exhaustion an also present higher rates of neurological toxicities (49). Strategies to mitigate exhaustion include reducing CD28 signaling strength through mutations in the CD3ζ or CD28 domains and modifying 4-1BB signaling (31). Interestingly, a recent long-term follow-up study found that long-lived CARs were predominantly CD4-positive, suggesting that this subset may be less prone to exhaustion and exhibit greater persistence (31).

Antigen modulation is the primary cause of CAR-T cells therapy resistance in B-cell malignancies. In pediatric and young adult B-ALL, approximately 50% of relapses are associated with CD19 loss (31, 50). Antigen density can be altered through CD19 mutations or deletions, alternative RNA splicing, lineage switching, epigenetic or post-transcriptional modifications, hyperglycosylation, antigen shedding and immune editing (48, 51–55). Novel receptors designed to lower the antigen density threshold are currently being developed, such as the expression of CCR binding to CD38 in combination with CAR showing activation at low antigen densities, effectively preventing antigen escape in preclinical models (32).

In 2016, Sotillo et al identified CD19 mutations and splice variants in pediatric B-ALL cases that relapsed after CD19 CAR-T therapy (51). Fischer et al also discovered that an alternatively spliced CD19 mRNA isoform missing exon 2, and thus the CART-19 epitope, but not isoforms missing the transmembrane and cytosolic domains, are present in leukemic blasts at diagnosis in children (56). Furthermore, the loss of SPPL3, causing hyperglycosylation of CD19, and its overexpression, resulting in the loss of CD19 (43). Changes in CD81 (52) and decreased expression of CD22 (57), CD19 transporter and BCR component respectively, have been observed in children and adults with relapsed B-ALL implicating evasion of tumor cells from CAR-T-mediated elimination.

Some patients who relapse after CD19-targeted therapy develop CD19-negative leukemia. To address this, a new approach involves the use of dual-targeting CAR-T cells against CD19/CD20 or CD19/CD22. The clinical trial NCT03330691, currently underway at Seattle Children’s Hospital (58) aims to provide valuable insights into this strategy.

Another significant mechanism of antigen loss is lineage switching, particularly in immature cell tumors such as ALL. This phenomenon has been noted in pediatric patients with B-ALL who relapsed with AML after CD19 CAR-T immunotherapy due to various genetic rearrangements and mutations (53, 59, 60). Anti-CD19 CAR-T cell therapy exerts selective pressure on CD19-expressing B-ALL, leading to tumor growth with different lineage markers and the loss of the CD19 target antigen (53, 60).

The prevalence of an ecological triad in leukemia, consisting of leukemia-initiating cells (LIC), inflammation, and TME, is proposed due to the inductive inflammation-tumorigenesis loop facilitated by an interconnecting microenvironment (61). TME has a crucial role in terms of response to cancer treatment, as it can attenuate the effectiveness of the infused CAR-T cells through several mechanisms (62). Recent work indicated that the loss of IFNGR1, JAK1 and JAK2 genes in the IFNγ receptor signaling pathway conferred resistance to CAR-T cell killing only in solid tumors but did not affect lymphoma or leukemia cell sensitivity to CAR-T cells (63). However, IFNγ has a dual function in CAR-T cell activity. Bailey et al. demonstrated that blocking IFNγ in CAR-T cells does not impair their cytotoxicity against leukemia cell lines. Paradoxically, it enhances their proliferation and reduces macrophage-mediated cytokines and chemokines associated with CRS. These findings suggest that IFNγ blockade may improve CAR-T cell function while reducing treatment-related toxicity in hematologic malignancies (64).

Myeloid-derived suppressor cells (MDSCs) have been shown to impair CAR-T cell efficacy by inhibiting their proliferation and cytolytic function. In mouse models, knocking down MDSCs with an anti-Gr-1 antibody significantly enhanced the anti-tumor activity of CAR-T cells. However, the absence of a specific marker for human MDSCs limits their targeted depletion using a single antibody (48). In hematological tumors, the exact involvement of multiple tumor-promoting cells such as MDSCs in CAR-T cells remains poorly understood. Additionally, while CAR-T therapy has demonstrated remarkable efficacy, it can also affect healthy cells, leading to off-target effects and toxicities. To mitigate these risks, a genetically encoded “suicide gene” has been developed, allowing for the selective elimination of transferred and genetically modified cells. This strategy serves as a safety switch, preventing unintended adverse effects and reducing therapy-related toxicity (33, 34).

Despite the great development of targeted cellular therapies, T-lineage neoplasms remain a challenge for CAR-T cells due to the limited ability to distinguish between therapeutic, normal, and neoplastic T-cells. Among patients with T-ALL, those with early T-cell progenitor (ETP)-ALL have a particularly poor response to remission induction chemotherapy and often require intensive chemotherapy and/or HSCT (65). Multiple target antigens shared between T effector cells and T-cell malignancies lead to CAR-T self-targeting, called fratricide, and to profound T-cell aplasia induced by the destruction of normal T-cells, leading to life-threatening opportunistic infections. CD7, CD5, TRBC1, CD38, and CD1a- targeted CAR-T cells have been developed and shown therapeutic potential (65). These antigens demonstrate limited fratricide and antileukemic activity *in vitro* and *in vivo* in xenograft models. Universal, fratricide-resistant CAR-T cells directed against the CD7 antigen, generated through CRISPR/Cas9 gene editing, have shown promising preclinical results (66, 67). Since CAR-T cells express CD7 on their surface, several approaches have been developed to mitigate the problem of fratricide by generating CD7-negative anti-CD7 CAR-T cells, including post-translational interference of CD7 expression and gene editing and the use of naturally occurring CD7-negative T cells (65).

### CAR-NK cells

Natural killer cells, known for their ability to eliminate tumor cells, have been identified as promising targets for engineering CAR-NK cells (68). CAR-NK cells complement CAR-T cells because they do not cause GVHD and can be derived from unrelated donors (69). Additionally, a real revolution in antitumor therapy is the inhibition of immune checkpoints, such as PD-1 and CTLA-4. Since PD-1 is expressed not only on tumor-associated T cells but also on NK cells, its blockade could allow NK cells to exert an effector role against HLA class I-deficient tumors that are undetectable by T cells (69). Additionally, several CAR constructs were engineered to recognize ALL and AML antigens such as CD19 (37, 70, 71), CD33 (71, 72), and CD123 (73, 74), incorporating a hinge region derived from CD34 to facilitate detection and selection of transduced NK cells.

Christodoulou et al. (73) developed NK cells with a CD123-targeting CAR and high IL-15 secretion capacity. The results demonstrated that these IL-15-modified CAR-NK cells maintained their anti-AML activity in a sustained manner, but this improvement in functionality was associated with significant systemic toxicities due to IL-15 secretion (73). While IL-15 production may contribute to systemic toxicity, other factors related to the genetic modification and CAR-NK cell activity could also play a role. In contrast, Caruso et al. (74) reported minimal systemic and endothelial toxicity with CAR.CD123-NK cells in mouse models, suggesting an improved safety profile compared to CAR.CD123-T cells and CAR.CD123-NK cells that secrete high levels of IL-15. These findings highlight the importance of considering multiple factors when assessing the toxicity and safety of CAR-NK cell-based therapies.

Researchers have focused on trogocytosis, which affects the effectiveness of CAR-NK cells by (1) reducing tumor antigen density, impairing target engagement, (2) induced self-recognition and continuous CAR-mediated engagement, resulting in fratricide of trogocytic antigen expressing NK cells (NKTROG+) and NK cell hyporesponsiveness (75). Functional status of NK cells is also relevant, since it has been described that their exhaustion directed by the leukemic microenvironment promotes malignant progression at the expense of antitumor responses (76).

Advancements in technology are automating the production of primary CAR-NK cells, despite initial challenges. Albinger et al. (72) used the CliniMACS Prodigy^®^ platform to generate large numbers of CD33-CAR-NK cells, which significantly reduced leukemic burden in a xenograft mouse model. This technology could facilitate clinical transitions for CAR-modified primary NK cells targeting various conditions (72).

Other methods are enhancing CAR-NK cells from hiPSCs, combined with immune checkpoint inhibitors in clinical trials for advanced solid tumors (70). Although still in progress for pediatric hematologic tumors, promising results are observed. NK cells can develop memory-like traits, with preactivated NK cells (IL-12, IL-15, and IL-18) showing improved memory and efficacy against AML. Current research focuses on mitochondrial health in NK cells, as dysfunction impairs immune responses (77).

Chimeric antigen receptor–natural killer therapies remain in the early stages of development compared to CAR-T therapies and have not yet been approved for clinical use in children. However, ongoing clinical trials suggest promising potential in pediatric populations. A notable example is the NCT03056339 clinical trial (78) led by Dr. Rezvani, which has shown good tolerability in adults and adolescents, supporting its potential as a basis for future pediatric clinical trials.

## Conclusion and future perspectives

Cell therapy has revolutionized the treatment of pediatric hematologic malignancies, with the most significant growth observed in resource-limited areas (79). Despite the success of HSCT and CAR-T cell therapy, challenges such as GVHD, relapses, antigen loss, and T-cell exhaustion persist. Future advancements will focus on refining HSCT through optimized donor selection, graft manipulation, and immune modulation while enhancing CAR-T efficacy via genetic engineering to secrete PD-1-blocking single-chain variable fragments (scFv) (80) or chimeric switch receptors to convert negative signaling into positive (81), metabolic reprogramming, and dual-targeting constructs such as Bivalent CAR, SynNotch and LINK CAR (31) against pediatric cancers.

**FIGURE 1:**
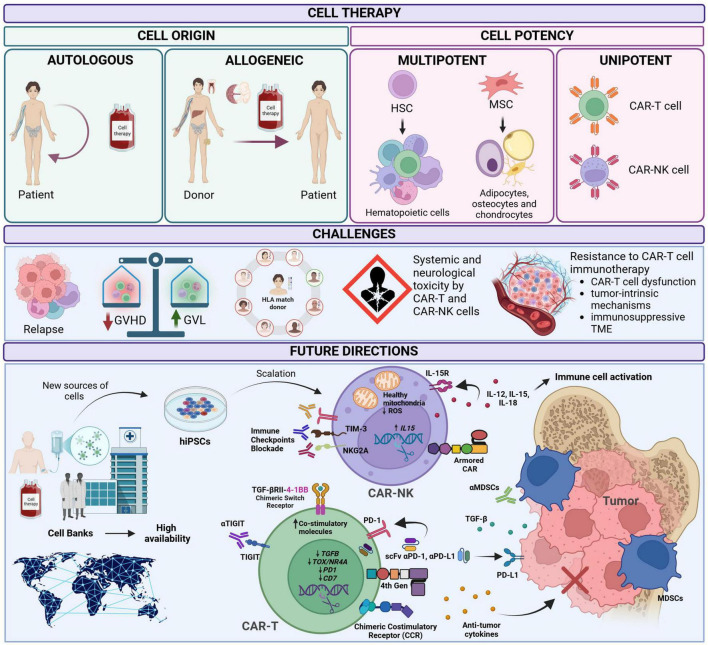
Cell therapy classification, challenges, and future directions. Classification based on cell origin and potency. Major challenges in cell therapy include relapses, the balance between graft-versus-host disease (GVHD) and graft-versus-leukemia (GVL) effects, systemic and neurological toxicity, and resistance to CAR-T cell immunotherapy. Future perspectives focus on expanding access in low- and middle-income countries through infrastructure development, advanced equipment, and specialized personnel; enhancing hematopoietic stem cell (HSC) expansion to optimize doses in hematopoietic stem cell transplantation (HSCT); increasing mesenchymal stromal cell (MSC) biobank availability; and improving CAR-T and CAR-NK cell. Created in BioRender. RAMIREZ, D. (2025) https://BioRender.com/i93y392.

Pioneering research led by women scientists has been instrumental in advancing cell-based immunotherapies, driving innovations in CAR-T and NK-cell engineering, and optimizing graft manipulation strategies. CAR-NK therapy offers a promising, off-the-shelf alternative with potent cytotoxicity and reduced toxicity, while strategies targeting the TME and leveraging CRISPR-driven precision medicine will further improve therapeutic outcomes. Overcoming accessibility barriers remains a critical priority, necessitating global collaborations and cost-effective innovations. Expanding access in low- and middle-income countries is particularly crucial given the high burden of acute leukemia and its associated mortality rates (82) (Figure 1). The future of cell therapy lies in harnessing engineered immune cell platforms, integrating artificial intelligence, and developing next-generation immunotherapies that redefine the standard of care for pediatric leukemia.
